# P-27. Impact of RSV( respiratory syncytial virus) Infection at ALM-VA

**DOI:** 10.1093/ofid/ofae631.234

**Published:** 2025-01-29

**Authors:** Ivanshi Gondalia, Jose Cadena-Zuluaga, Delvina Ford

**Affiliations:** University of Texas at San Antonio, San Antonio, Texas; University of Texas health and science center San Antonio, Audie L. Murphy VA Medical Center, San Antonio, Texas; South Texas Veterans Health Care System, San Antonio, Texas

## Abstract

**Background:**

RSV infections, known for their respiratory complications, present a unique challenge in healthcare settings. Respiratory syncytial virus (RSV) causes substantial morbidity and mortality in older adults, resulting in approximately 60,000–160,000 hospitalizations and 6,000–10,000 deaths annually among adults aged ≥65 years

**Methods:**

Retrospective review of the RSV cases from January 1, 2022 to October 27, 2023, at the South Texas Veterans healthcare system. We collected data(age and outcome of patients) from Theradoc (infection control surveillance tool used for surveillance) on positive RSV patients seeking care at South Texas Veterans Health Care System including the age of the positives, whether they had respiratory symptoms, were admitted to the hospital, and whether this admission was related to the respiratory symptoms.

**Results:**

130 patients with positive RSV( by Biofire or Fluvid RSV PCR) were studied. The mean age of the population was 61.7 years, with a standard deviation of 15.8 and a median of 63.

Out of these, 73.8%(96/130) had only upper respiratory symptoms( Per CDC definition includes: rhinorrhea, cough, sneezing, nasal congestion) and 17.6% (23/130) had lower +/- upper respiratory symptoms.( Lower respiratory symptoms were defined as Tracheobronchitis, pneumonia, exacerbation of COPD, asthma, exacerbation of cardiac or neurological illness)

21.5%(28/130) of them were admitted due to some other reason but were found to be positive for RSV and 10.7%(14/130) of them were admitted due to RSV.

Almost 61% (79/130)of them were aged 60 and above and out of those, RSV was the reason to seek care in 83.5%(66/79) of them. 83.3% (55/66) of them had upper respiratory symptoms and almost 26% of them had lower +/-upper respiratory symptoms.

Almost 30%(23/79) of the patients over the age of 60 were admitted and RSV was the reason for admission in 53% (14/23) of them.

Variables included the age of the positives, whether they had respiratory symptoms, were admitted to the hospital, and whether this admission was related to the respiratory symptoms

**Conclusion:**

This project's findings will contribute to promoting the vaccination against RSV in patients older than 60 years of age and improving patient outcomes.

**Disclosures:**

**All Authors**: No reported disclosures

